# Electromagnetic Interference Shielding Properties of Conductive Polyaniline/TiO_2_/MoS_2_ Hybrid Composites

**DOI:** 10.1021/acsomega.5c02527

**Published:** 2025-06-23

**Authors:** Aravinth Dhanasekaran, Amit Malakar, A Lakshmanan, Vitezslav Stranak, Suryasarathi Bose, Kannadassan Dhanaraj, Velmurugan Venugopal

**Affiliations:** † School of Advanced Sciences, 30026 Vellore Institute of Technology, Vellore, Tamil Nadu 632014, India; ‡ Department of Materials Engineering, 29120 Indian Institute of Science, C.V. Raman Avenue, Bangalore 560012, India; § Faculty of Science, 48271 University of South Bohemia, Branisovska 1760, Ceske Budejovice 37005, Czech Republic; ∥ School of Electronics Engineering, Vellore Institute of Technology, Vellore, Tamil Nadu 632014, India

## Abstract

This study investigates the hybrid ternary composite of polyaniline (PANI), titanium dioxide (TiO_2_), and molybdenum disulfide (MoS_2_) to improve electromagnetic interference (EMI) shielding in high-frequency electronic devices. The study examines the shielding effectiveness (SE) of this hybrid composite by altering MoS_2_ concentrations. The composite was created through a four-stage in situ polymerization method, with MoS_2_ levels at 10, 20, and 30 wt % and a fixed TiO_2_ content of 10 wt %. A Vector network analyzer (VNA) was employed to measure EMI SE and complex permittivity in the 8.2–12.4 GHz frequency range. The composite with 20 wt % MoS_2_ exhibited the highest SE of 50.86 dB for 3 mm thickness, surpassing other formulations. The enhanced shielding performance is credited to the synergistic effects of MoS_2_ and TiO_2_, which enhance the impedance matching, electrical conductivity, and electromagnetic wave dissipation. Further analysis revealed that factors, such as binding and agglomeration during in situ polymerization, along with electromagnetic absorption and multiple scattering mechanisms, significantly influence the shielding process. The results indicate that the optimized PANI/TiO_2_/MoS_2_ hybrid composite shows promise as an effective EMI shielding material for cutting-edge electronic and communication applications.

## Graphical abstract

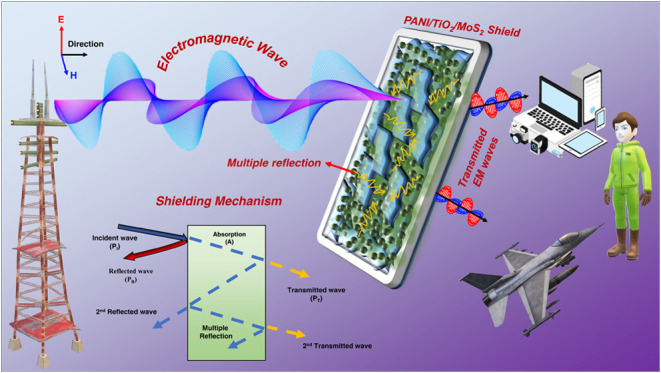

## Introduction

The wide range of applications for electromagnetic (EM) waves in the megahertz (MHz) to gigahertz (GHz) range has become an essential component of contemporary technology. Electromagnetic interference (EMI), which affects signal transmission and the effectiveness of electronic systems, is one of the major problems of application in this frequency range. As a type of electromagnetic pollution, this interference can cause device malfunctions as well as possible health risks like tissue damage, cancer, depression, and headaches.^1–4^ To counter these effects, effective EMI shielding strategies are essential for ensuring reliable performance and safety in advanced technological environments.

The X-band (8.0–12.0 GHz) holds particular relevance due to its use in radar systems, satellite communications, wireless networks, remote sensing, and radio astronomy.^5,6^ Under specific exposure circumstances, X-band frequencies may have biological effects. Despite being classified as nonionizing radiation, prolonged exposure to high power levels can cause thermal effects, including tissue heating, which may cause structural and cellular damage. Particularly, there is a risk for cataract development and high-intensity radiation burns to the eyes and skin. Although conclusive evidence is still being investigated, prolonged low-power exposure has also been linked to oxidative stress, changes in cellular function, and gene expression. These hazards show how strict regulations are required to reduce human exposure to electromagnetic fields.^7,8^

In order to protect the electronic systems and human health, EMI shielding materials attenuate or reflect the electromagnetic waves. The material’s capacity to prevent radiation penetration while maintaining performance and durability determines the shielding effectiveness (SE). In the electrical, electronics, and telecommunications sectors, where people are exposed to electromagnetic radiation on a daily basis, EMI shielding is especially important. Metals and other conductive materials have long been preferred due to their excellent shielding and reflective properties.^9,10^ However, their thickness, weight, and cost have driven the search for alternative materials that are lightweight, cost-effective, and capable of neutralizing GHz-range waves.

High-performance EMI shielding materials must exhibit excellent electrical conductivity and magnetic properties to enhance the reflection and absorption mechanisms. Ferrimagnetic and ferromagnetic materials, along with metallic conductors, are commonly employed to achieve this balance.^11,12^ However, with increasing EM wave frequencies and stricter global regulations, the need for advanced materials that combine conductivity, flexibility, and environmental sustainability has become critical. Recent advancements in nanomaterials, particularly hybrid composites, offer promising solutions to these challenges. By integrating properties such as lightweight design, enhanced tunability, and superior shielding performance across broad frequency ranges, these materials address the evolving demands of modern EMI shielding applications. Their potential to protect sensitive equipment while mitigating health risks underscores their significance in the development of next-generation shielding technologies.^8^

EMI shielding through absorption requires the shielding material to possess electric and magnetic dipoles capable of interacting with the electric (*E*) and magnetic (*H*) fields of the incident electromagnetic radiation. These dipoles can be provided by materials with a high dielectric constant, such as ZnO, SiO_2_, TiO_2_, or BaTiO_3_, or materials with high magnetic permeability, including carbonyl iron, Ni, Co, Fe metals, γ-Fe_2_O_3_, NiFe_2_O_4_, and Fe_3_O_4_. However, these materials or their composites face challenges such as low permittivity or permeability at GHz frequencies, added weight, limited performance across a narrow frequency band, and difficulties in processing. Additionally, poorly dispersed compositions may lead to gaps in the host matrix that are devoid of filler material, resulting in radiation leakage and a reduction in the shielding efficiency.^13–20^

Molybdenum disulfide (MoS_2_) is a two-dimensional material with a graphene-like layered structure. Its unique physical, chemical, and mechanical properties have drawn significant attention from researchers in recent years. MoS_2_ also exhibits notable dielectric loss and low density, making it an ideal candidate for lightweight electromagnetic absorbers. One of its morphological features is the blooming flower-like structure formed by stacked flakes, which increases the specific surface area and enhances its ability to absorb electromagnetic waves.^21–23^ Conducting polymers with a finite conductivity and the ability to block microwaves present a promising solution in this context. Specifically, synthetic metals such as poly­(aniline) (PANI)-based compositions have garnered significant interest due to their tunable conductivity, adjustable permittivity or permeability, low density, resistance to corrosion, affordability, and excellent thermal and environmental stability. These properties make them highly suitable for a wide range of technological and commercial applications.^10^

While binary composites like PANI/MoS_2_, TiO_2_/MoS_2_, and PANI/TiO_2_ have been explored in prior studies,^24–27^ the ternary PANI/TiO_2_/MoS_2_ system introduces a multi-interface architecture that enhances both dielectric and conductive loss mechanisms. In EMI shielding, achieving a balance between reflection and absorption is essential for achieving high SE. Conductive materials like PANI enhance reflection by reducing the skin depth, whereas materials such as TiO_2_, with a high dielectric constant and moderate conductivity, enable greater wave penetration, enhancing absorption. Nevertheless, TiO_2_ has been considered a potential shielding component by virtue of its excellent safety, eco-friendliness, and low cost.^28^ Additionally, the MoS_2_ layered structure plays a significant role in further improving absorption due to its excellent dielectric loss, unique morphology, and low density. Combining MoS_2_, TiO_2_, and PANI in a composite effectively balances these mechanisms while ensuring proper impedance matching with free space. Despite their potential, studies focusing on the ternary PANI/TiO_2_/MoS_2_ composite for EMI shielding applications are still scarce.

This work investigates the EMI shielding capabilities of the PANI/TiO_2_/MoS_2_ (PTM) composite, synthesized through an in situ polymerization process. The PTM composite exhibits excellent shielding performance across the X-band frequency range. Our study focuses on the impact of varying MoS_2_ weight percentages and sample thicknesses on the shielding effectiveness. Detailed analyses of the prepared samples, including their morphology and electromagnetic properties, were conducted using advanced characterization techniques. The innovative ternary PANI/MoS_2_/TiO_2_ composite demonstrates a well-integrated structure with improved dielectric and electrical properties, making it a strong candidate for next-generation EMI shielding technologies.

## Materials and Method

Aniline monomer was procured from Sigma-Aldrich, ammonium persulfate (APS) and molybdenum disulfide of 98% purity were purchased from Sisco Research Ltd., and hydrochloric acid (HCl) was purchased from Avra Synthesis Pvt. Ltd.

### PANI Synthesis

For the typical polyaniline synthesis,^29^ aniline (10 mL, 0.1097 mol) was dissolved in a 1 M HCl solution (1 mL, 100 mL) and the solution was cooled to 0 °C. The oxidizing agent was prepared by dissolving ammonium persulfate (1.25 g, 0.0548 mol) in HCl solution (1 M, 100 mL). The aniline solution was vigorously stirred at 0 °C before that, and then the oxidant solution was added drop by drop. A green precipitate emerged within 10–15 min. The reaction mixture was stirred for 4 h at 0 °C and for 20 h at room temperature. Afterward, 200 mL of water was added to the reaction mixture to setttle the precipitation. The unreacted monomer was removed from the precipitation by washing it with DD water and 1 M HCl solution. The collected green precipitate was washed with DD water until the washings became colorless.

### PANI/TiO_2_ Composite

Ten wt % TiO_2_ was added to Monomer solution (aniline dissolved 1 M HCl solution), later the solution was sonicated for an hour using ultra probe sonicator at 20 kHz. After that, the solution was cooled to 0 °C. Then, the oxidizing agent was added for the polymerization process, which was explained in the previous section. The sample code was named as PT for TiO_2_/PANI composite.

### PANI/TiO_2_/MoS_2_ Composite

MoS_2_ and TiO_2_ were added to the monomer solution (aniline dissolved HCl solution) in a 1:1 ratio (10 wt %/10 wt %). The solution was sonicated at 20 kHz for an hour using an ultraprobe sonicator. Subsequently, this solution was cooled to a temperature of 0 °C. Following this, the polymerization process was initiated by the addition of an oxidizing agent, as detailed in the PANI synthesis section. The detailed synthesis protocol of PANI/TiO_2_/MoS_2_ hybrid composite is shown in Figure 1. MoS_2_ and TiO_2_ materials were prepared in a variety of weight ratios, including 1:1, 2:1, 3:1, (10 wt %/10 wt %), (20 wt %/10 wt %), and (30 wt %/10 wt %). The sample code is followed by PTM1, PTM2, and PTM3, respectively.

**Figure 1:**
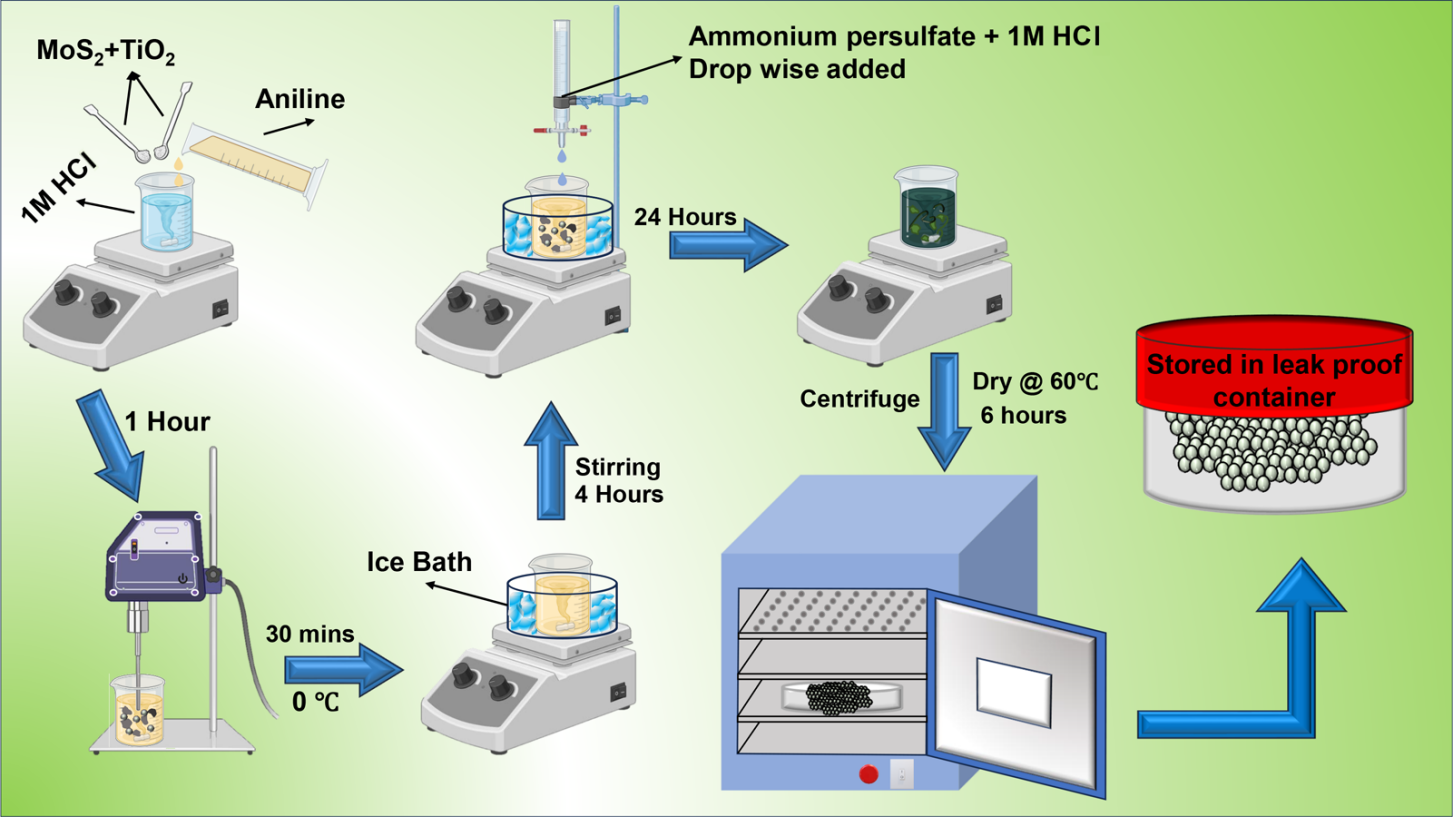
Synthesis procedure of PANI//TiO_2_/MoS_2_ (Created in BioRender https://BioRender.com/gsmi5my).

## Characterization

Structural properties of the prepared composite samples were studied using an X-ray diffractometer (XRD) (Bruker, Germany, Model: D8 advance) by Cu Kα radiation of wavelength 1.5406 Å in the 2Θ range of 5–80°. Microstructural analysis was performed using a Field emission scanning electron microscope (FE-SEM) Thermo Fisher FEI QUANTA 250 FEG. Raman spectroscopy analysis was conducted utilizing a Wi-Tec α 300 R.A. Spectrometer equipped with an argon laser (wavelength λ = 536 nm, output power 20–50 mW). Electromagnetic Interference Shielding Effectiveness and dielectric properties were measured using a Keysight Technologies N9918A vector network analyzer (VNA) within the frequency range of 8.2–12.4 GHz (X-band).

## Results and Discussion

### Chemical Structure Characterization

XRD is employed to confirm the crystalline structure of the composite and to ensure that the commercial MoS_2_ and TiO_2_ are successfully incorporated into the PANI matrix. Figure 2 shows the XRD patterns of the synthesized PANI, PT (PANI/TiO_2_) and PTM (1–3) (PANI/TiO_2_/MoS_2_(different weight percentages of MoS_2_)) composites. The synthesized PANI shows distinct broad peaks at 14.75° (001), 20.31° (020), and 25.23° (200) that correspond to the periodicity parallel to the polymer backbone and is associated with the spacing between polymer chains. This broad peak is characteristic of the semicrystalline regions in PANI and results from the π–π stacking interactions between adjacent polymer chains.^30^ After incorporating TiO_2_ nanoparticles (10 wt %) into the PANI matrix, which can disrupt the π–π stacking interactions between polymer chains, thus leading to a significant disruption of long-range order. Additionally, the crystalline form of TiO_2_ gives a strong and sharp peak, which overshadows the weaker polymer peaks. The peaks observed at 25.59° (101), 38.14° (004), 48.28° (202), 55.28° (211), 62.93° (204), 69.15° (116), 70.65° (220) and 75.44° (215) are responsible for the TiO_2_ anatase phase (compared with JCPDS card number 21-1272).^31,32^ Also, a weak TiO_2_ rutile phase peak was observed at 27.73, 36.31, and 54.15° which correspond to (110), (101) and (210), respectively (compared with JCPDS card number 21-1276).^33^ This confirms that TiO_2_ majorly consists of the anatase phase.

**Figure 2:**
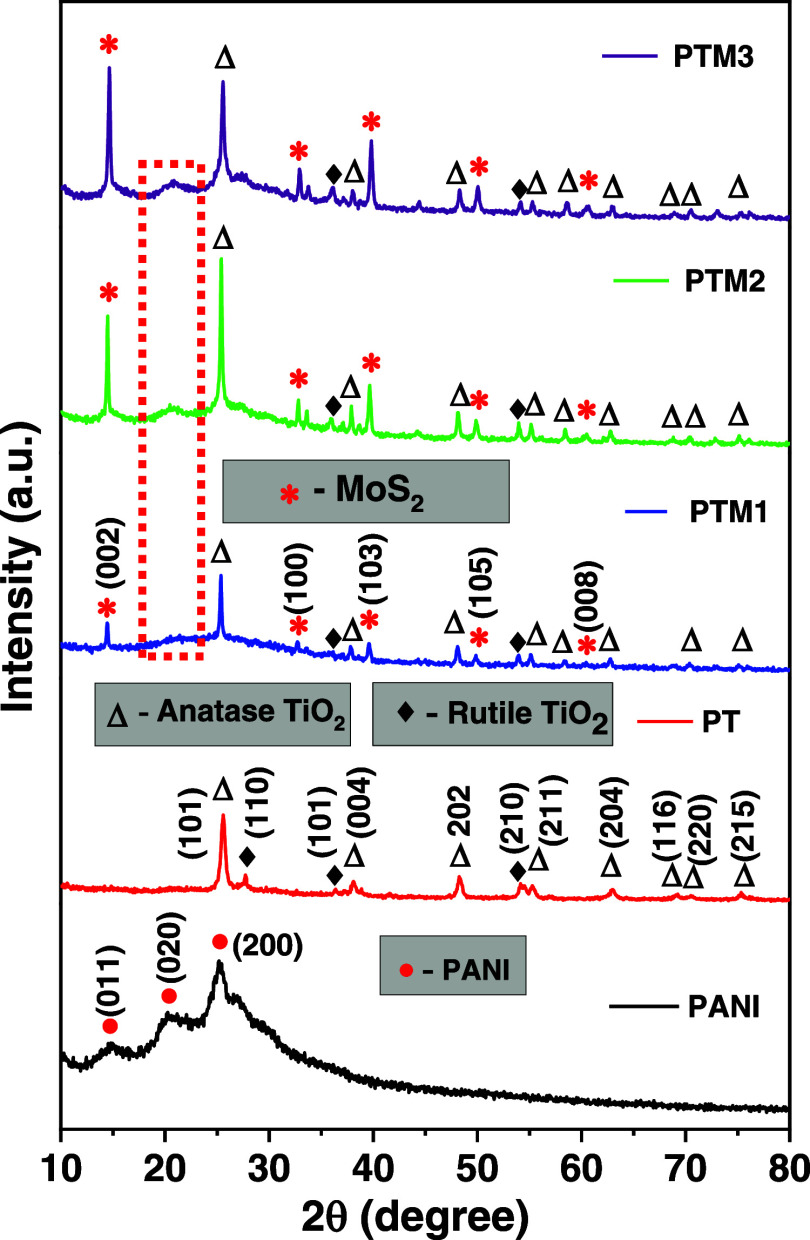
XRD spectra of pure PANI, PANI/TiO_2_ (PT) and PANI/TiO_2_/MoS_2_ (PTM) in various MoS_2_ concentrations.

Further adding MoS_2_ nanosheets (10 wt %) into the PANI/TiO_2_ composite, the signature peaks of rhombohedral MoS_2_ were observed at 14.66° (002), 32.93° (008), 39.81° (103), 50.06° (105) and 60.61° (008) (compared with JCPDS card number 06–0097).^34^ Also, the PANI broadened peaks started to appear in the range of ∼20°. As the concentration of MoS_2_ increases to 20 wt % and then to 30 wt %, the intensity of the MoS_2_ peaks grows stronger. At the same time, the intensity of PANI also increases. This indicates that the layered morphology of MoS_2_ may provide a templating or aligning effect, enhancing more organized stacking within the PANI matrix. Otherwise, the presence of MoS_2_ may reduce the relative dominance of TiO_2_ peaks, making the PANI peaks discernible.

These XRD results confirm the successful formation of PANI/TiO_2_/MoS_2_ composites without any secondary phases.

Raman spectroscopy is used to validate the presence of specific materials such as MoS_2_, TiO_2_, and PANI and to detect any structural changes or interactions within the composite. Figure 3 shows the Raman spectra for (a) PANI, (b) PT and (c) PTM1 samples. The PANI sample exhibits Raman peaks at 1158, 1362, 1451, 1584, and 1608 cm^–1^. The peak at 1158 cm^–1^ corresponds to C–H stretching of the semiquinoidal ring, the peak at 1362 cm^–1^ is associated with delocalized polaronic C–N^+^ stretching, the peak at 1451 cm^–1^ relates to C–N stretching of the benzenoid ring, the peak at 1584 cm^–1^ indicates C = N stretching of the quinoid ring and the peak at 1608 cm^–1^ is attributed to C–C stretching of the benzenoid ring. This confirms the formation of conductive PANI.^35^ After incorporating TiO_2_ into the PANI matrix, the well-defined crystalline structure of TiO_2_ suppressed the intensity and broadened the peaks associated with PANI. The peaks observed at 147 cm^–1^ (Eg mode), 198 cm^–1^ (Eg mode), 503 cm^–1^ (A1g mode), and 633 cm^–1^ (Eg mode) are attributed to the anatase phase of TiO_2_.^32^ Here, Eg modes are generated by the vibrations of Ti–O bonds, while A1g modes are produced by the vibrations of O–Ti–O bonds. Further, there is no indication of a peak for the rutile phase since the rutile phase present in the TiO_2_ sample is very minimal. These findings are further supported by the XRD results. Likewise, after adding MoS_2_ into the composites, we noticed additional peaks at 380 and 403 cm^–1^, which are associated with MoS_2_. The peak at 380 cm^–1^ is due to E_2*g*_^1^ mode and the peak at 403 cm^–1^ is due to A_1g_ mode.^35^ Additionally, the peaks of PANI become sharper rather than broader after the addition of MoS_2_. This may be due to layered morphology offering a stabilizing the PANI matrix.^35^ A similar trend was observed in the XRD results of all the PTM samples.

**Figure 3:**
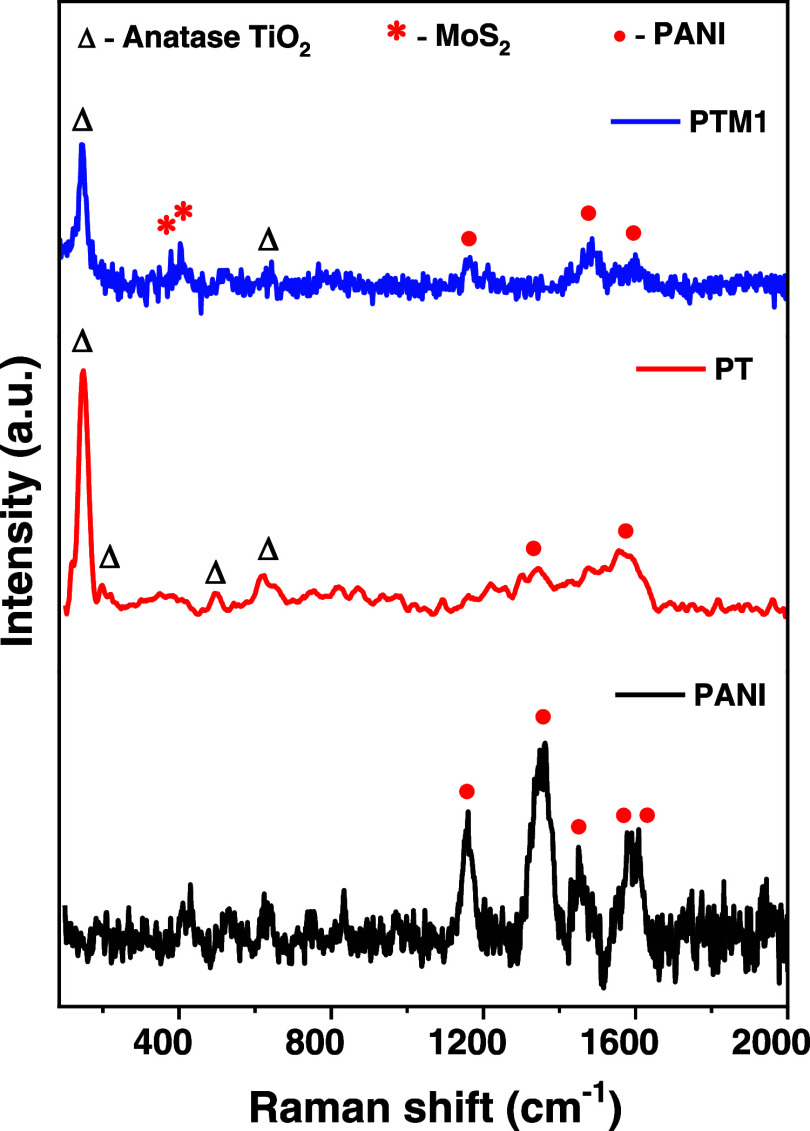
Raman spectra of pure PANI, PANI/TiO_2_(PT), and PANI/TiO_2_/MoS_2_ (PTM1) composites.

All the Raman spectra confirm that our composite does not form any secondary phases and the XRD results also support this finding.

### Microstructural Characterization

FESEM is employed to investigate the surface morphology and microstructure of the hybrid composite. Figure 4 illustrates the FESEM images of pure PANI, as well as the composites PT and PTM1. The PANI sample exhibits a fibrous and granular morphology, featuring interconnected polymer chains that form a porous structure (Figure 4a). When TiO_2_ is incorporated into the PANI matrix, the surface becomes more textured and heterogeneous (Figure 4b). Distinct TiO_2_ particles are embedded within the PANI matrix, giving it a rougher appearance compared to pure PANI. The inclusion of MoS_2_ in the PANI/TiO_2_ system introduces layered nanosheets, which are clearly visible in the FESEM image (Figure 4c). These nanosheets are distributed throughout the matrix, resulting in a hierarchical structure. Overall, the morphology becomes increasingly complex, combining fibrous, particulate, and layered characteristics. From this, the layer morphology of MoS_2_ enhances the PANI matrix chains, which was confirmed by XRD results. Further, EDS spectra were recorded for the PTM1 composites to determine their elemental composition (Figure 5a). The elemental composition, represented as weight percentages, is as follows: Carbon (C): 51.66%, Nitrogen (N): 13.04%, Titanium (Ti): 7.10%, Oxygen (O): 15.05%, Molybdenum (Mo): 6.85%, and Sulfur (S): 6.30%. The corresponding elemental mapping confirms that all elements are distributed uniformly in the PANI matrix (Figure 5b–g).

**Figure 4:**
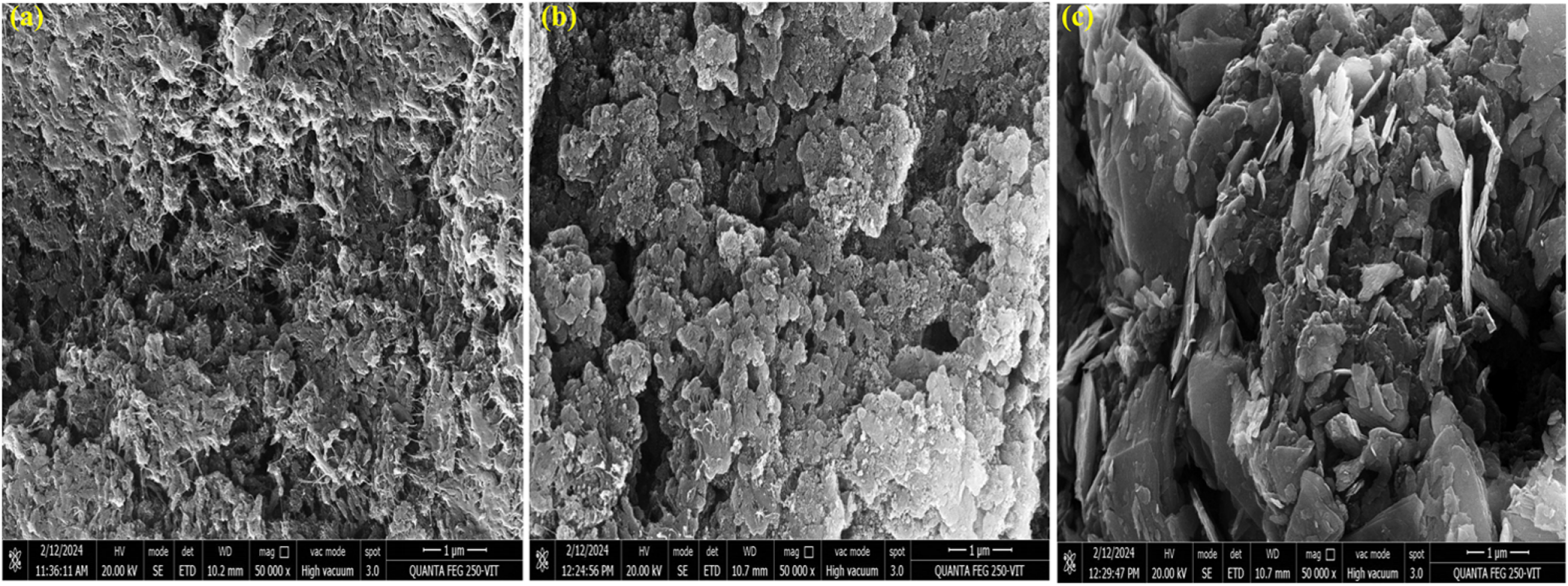
FESEM Images of (a) PANI, (b) PANI/TiO_2_(PT), and (c) PANI/TiO_2_/MoS_2_(PTM1).

**Figure 5:**
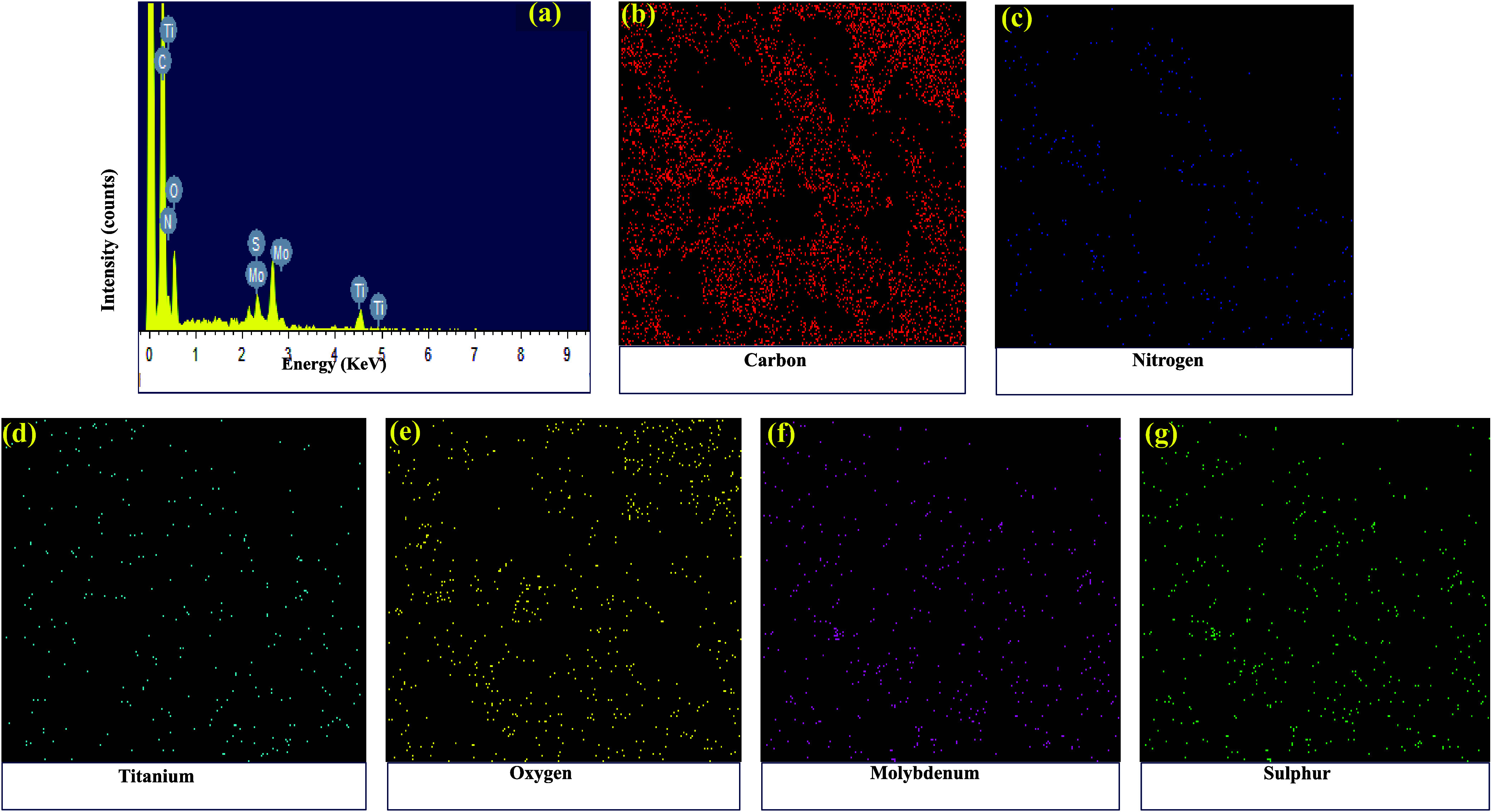
(a) EDS spectra and (b) to (g) is EDS mapping of PANI/TiO_2_/MoS_2_ (PTM1) composite.

### Electromagnetic Shielding Effectiveness Characterization

Two-port scattering parameters were measured across the X-band frequency range. Rectangular composite pellets were fabricated under a pressure of 2000 psi to conform to the dimensions of the X-band waveguide (22.86 mm × 10.16 mm) and positioned on a WR90 waveguide. The composite-loaded waveguides were connected to the vector network analyzer (VNA) via coaxial cables. Each composite was prepared in three thicknesses: 1.25, 1.75, and 3 mm. The 1.25 mm thickness samples are coded as PT-1, PTM1-1, PTM21, PTM31; 1.75 mm thickness samples are coded as PT-2, PTM1–2, PTM2–2, PTM3–2; while the 3 mm thickness samples are coded as PT-3, PTM1–3, PTM2–3, PTM3–3, respectively. The compositions are mentioned in the section Materials and Methods.

When a material interacts with an EM wave at microwave frequencies, its capacity to shield the wave is quantified as electromagnetic interference shielding effectiveness (EMI SE). This effectiveness is primarily determined by three components: Reflectivity (*R*), Absorptivity (*A*), and Correction factor (*M*). *R* represents the reflection coefficient, while *A* indicates the absorption coefficient. The correction factor (*M*) accounts for multiple internal reflections within thin, finite-dimensional media. These parameters describe the reflection, absorption, and transmission phenomena in shielding materials, which collectively influence the transmittivity or transmission coefficient (*T*).^36,37^ The total shielding effectiveness (SE_T_) is defined based on these factors

1 $$SE_{T}=SE_{R}+SE_{A}+SE_{M}$$

Reflection efficiency (SE_R_), multiple reflection efficiency (SE_M_), and absorption efficiency (SE_A_) are expressed in decibels (dB) as log-scale representations of *R*, *M*, and *A*, respectively. To evaluate the electromagnetic shielding effectiveness (EM SE) using a VNA with a symmetric two-port network model, the scattering parameters (*S*_11_, *S*_12_, *S*_21_, and *S*_22_) were measured across the X-band frequency range. These parameters are critical for determining the transmission (*T*) and reflection (*R*) coefficients using the following equations

2 $$\begin{array}{l} R=\left|S_{11}{|}^{2}=\right|S_{22}{|}^{2}\\ T=\left|S_{21}{|}^{2}=\right|S_{12}{|}^{2}\\ A=1-R-T \end{array}$$

The following equations can then be used to determine the values of SE_R_ and SE_A_.

3 $$SE_{R}=10\;\log\frac{1}{\left(1-|S11{|}^{2}\right)}$$

4 $$SE_{A}=10\;\log\frac{\left(1-|S11{|}^{2}\right)}{|S11{|}^{2}}$$

5 $$SE_{T}=10\;\log\frac{1}{|S12{|}^{2}}=10\;\log\frac{1}{|S21{|}^{2}}$$

and the effective absorption coefficient

6 $$A_{\mathrm{eff}}=\frac{1-R-T}{1-R}$$

Generally, *A*_eff_ becomes significant when the reflection shielding effectiveness (SE_R_) exceeds 10 dB, and multiple internal reflections (SE_M_) are neglected. The overall shielding effectiveness (SE_T_) is subsequently calculated by combining SE_R_ and SE_A_ as

7 $$SE_{T}=SE_{R}+SE_{A}=10\;\log\left(1/T\right)$$

The study of PANI/TiO_2_/MoS_2_ composites with varying MoS_2_ concentrations and sample thicknesses provides critical insights into their EMI shielding performance and dielectric properties, which can be analyzed through Debye theory. According to Debye theory, the complex permittivity of a material is expressed as

8 $$\varepsilon_{r}=\varepsilon^{\prime }-j\varepsilon^{\prime \prime }$$

where ε′(ω) is the real part, representing the material’s ability to store energy, and ε″(ω) is the imaginary part, representing the energy dissipation. The relationship between real and imaginary permittivity is governed by the equations

9 $$\varepsilon^{\prime }\left(\omega \right)=\varepsilon_{\infty }+\frac{\varepsilon_{s}-\varepsilon_{\infty }}{1+\omega^{2}\tau^{2}}$$

10 $$\varepsilon^{\prime \prime }\left(\omega \right)=\frac{\left(\varepsilon_{s}-\varepsilon_{\infty }\right)\omega \tau }{1+\omega^{2}\tau^{2}}+\frac{\sigma }{\omega \varepsilon_{0}}$$

where ε_0_ is the vacuum permittivity, ε_s_ is the static permittivity, ε_∞_ is the high frequency permittivity, ω = 2π*f* is the angular frequency, τ is the relaxation time, and σ is the bulk electrical conductivity. The Nicolson–Ross–Weir method was used to compute the real and imaginary parts of complex permittivity from measured *S*-parameters, allowing for a detailed analysis of the material’s dielectric behavior and EMI SE_T_.

From the measured results at 8.2–12.4 GHz frequency, first, the contributions of the above-mentioned parameters are calculated and presented in Figure 6. The corresponding EMI SE values are 8.00, 24.86, 30.92, 22.40, 26.63, 45.63, 30.97, 32.99, 50.86, 30.50, 40.34, and 44.21 dB for the PT-1, PT-2, PT-3, PTM1-1, PTM1-2, PTM1-3, PTM2-1, PTM2-2, PTM2-3, PTM3-1, PTM3-2 and PTM3-3, respectively. All samples exhibit excellent EMI shielding capabilities, surpassing the minimum requirement of >20 dB for commercial applications.

**Figure 6:**
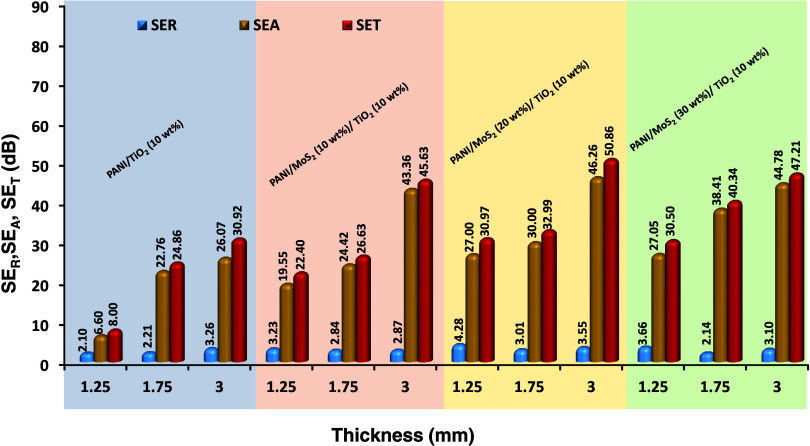
Contribution of absorption and reflection to the total SE.

Figure 7 shows 1.25 mm thick samples. The control PANI/TiO_2_ composite (PT1) demonstrated an initial SE_T_ of 8 dB, which is considered to be low and indicates poor EMI shielding capability. The real and imaginary permittivity values remained relatively constant, showing moderate dielectric polarization and minimal energy loss, reflecting the insulating nature of the base composite.

**Figure 7:**
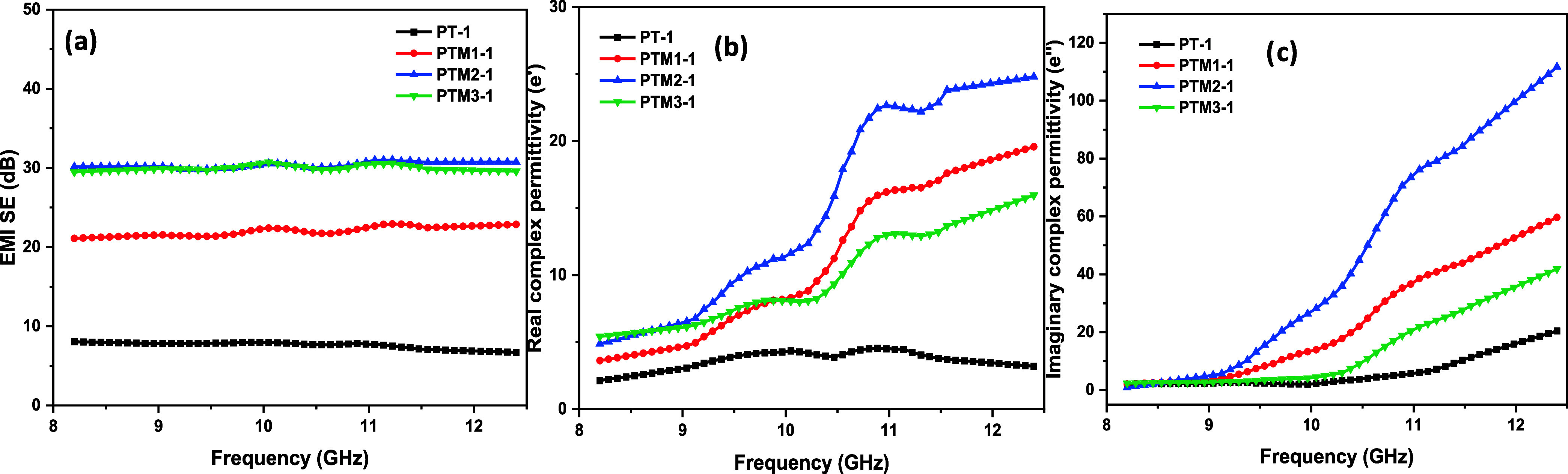
EMI SE and dielectric properties of PANI/MoS_2_/TiO_2_ and PANI/TiO_2_: (a) SE_T_ of the 1.25 mm thick PANI/TiO_2_ and PANI/MoS_2_/TiO_2_ composites, (b, c) complex real and imaginary part of permittivity of the 1.25 mm thick PANI/TiO_2_ and PANI/MoS_2_/TiO_2_ composites.

The addition of 10 wt % MoS_2_ to the composite (PTM1-1) resulted in a high SE_T_ of 22.40 dB, indicating a significant enhancement in shielding effectiveness. The real part of permittivity increased, suggesting improved polarization within the material. A slight rise in the imaginary part of permittivity was also observed, indicating enhanced energy dissipation, likely due to the interaction between MoS_2_ and the polymer matrix, which facilitated greater energy loss at the applied frequencies. This SE_T_ increase aligns with Debye theory, as the introduction of MoS_2_ enhances the material’s dielectric properties, leading to improved polarization and energy dissipation.

Increasing the MoS_2_ content to 20 wt % (PTM2-1) led to a higher SE_T_ of 30.97 dB, a considerable improvement over the PTM1-1 composite. This indicates a substantial enhancement in the composite’s shielding effectiveness, as the additional MoS_2_ likely contributed to stronger polarization and dielectric losses. The increased imaginary permittivity at higher frequencies indicates improved energy dissipation, while the steady increase in real permittivity suggests enhanced energy storage capacity. However, when 30 wt % MoS_2_ (PTM3-1) was incorporated, the SE_T_ remained at 30.50 dB, suggesting that excessive MoS_2_ content may result in agglomeration or inhomogeneity within the composite. This agglomeration could impede effective interaction between MoS_2_ and PANI, limiting further improvements in shielding effectiveness.

In the case of 1.75 mm thick specimens shown in Figure 8, the control PT2 composite exhibited a SE_T_ of 24.86 dB. The real component of permittivity showed a consistent increase across the frequency spectrum, while the imaginary component remained relatively stable. This suggests that the thicker composite demonstrated moderate shielding capabilities, with limited energy dissipation throughout the frequency range.

**Figure 8:**
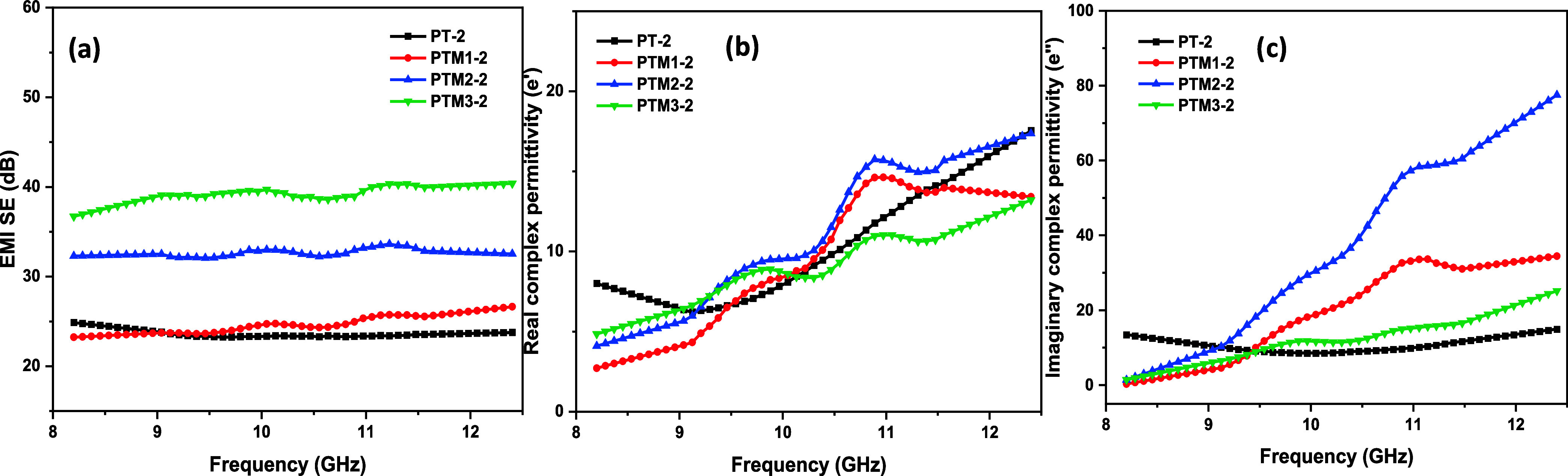
EMI SE and dielectric properties of PANI/MoS_2_/TiO_2_ and PANI/TiO_2_: (a) SE_T_ of the 1.75 mm thick PANI/TiO_2_ and PANI/MoS_2_/TiO_2_ composites, (b, c) complex real and imaginary part of permittivity of the 1.75 mm thick PANI/TiO_2_ and PANI/MoS_2_/TiO_2_ composites.

The addition of 10 wt % MoS_2_ (PTM1–2) resulted in an improved SE_T_ of 26.63 dB. A gradual rise in real permittivity was observed, indicating enhanced energy storage capacity. Simultaneously, the imaginary permittivity began to increase at higher frequencies, pointing to improved energy dissipation. This trend was further amplified with 20 wt % MoS_2_ (PTM2–2), which elevated the SE_T_ to 32.99 dB and further enhanced both real and imaginary permittivity components. These observations suggest that higher concentrations of MoS_2_ led to improved polarization and dissipation at elevated frequencies, consequently enhancing EMI shielding performance.

Adding filler beyond the percolation threshold often results in a significant change in the composite’s electrical conductivity, primarily due to possible agglomeration or uneven filler distribution. In our research, at 30 wt % MoS_2_ (PTM3–2), the SE_T_ achieved 40.34 dB, suggesting nearly optimal shielding performance. However, increasing the concentration beyond this point did not significantly enhance the SE_T_, and the dielectric properties began to exhibit signs of saturation. Although no direct morphological evidence of MoS_2_ agglomeration was detected, this behavior might be due to the potential for MoS_2_ aggregation, which disrupts uniform dispersion and diminishes shielding effectiveness. This aligns with percolation theory, where excessive filler content can result in nonuniform material properties and saturation in EMI shielding performance.^38,39^

The 3 mm thick PT3 samples exhibited a SE_T_ of 30.92 dB in Figure 9, suggesting moderate EMI shielding capabilities with restricted energy dissipation across the measured frequency spectrum. When 10 wt % MoS_2_ was added (PTM1–3), the SE_T_ increased to 45.63 dB, indicating enhanced dielectric polarization and energy dissipation, consistent with Debye theory principles. The composite displayed improved dielectric response due to the cooperative interaction between PANI and MoS_2_. Increasing MoS_2_ content to 20 wt % (PTM2–3) resulted in a SE_T_ of 50.86 dB, marking a substantial improvement in EMI shielding performance. This enhancement was attributed to superior energy storage and dissipation at higher frequencies, demonstrating robust dielectric behavior and effective shielding ability.

**Figure 9:**
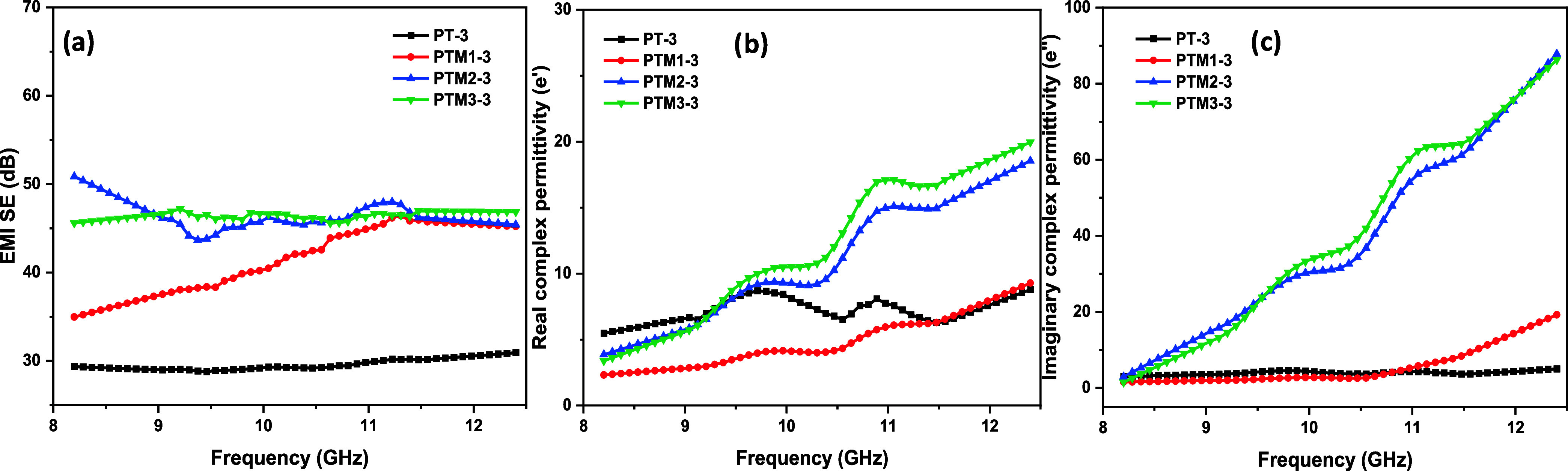
EMI SE and dielectric properties of PANI/MoS_2_/TiO_2_ and PANI/TiO_2_: (a) SE_T_ of the 3 mm thick PANI/TiO_2_ and PANI/MoS_2_/TiO_2_ composites, (b, c) complex real and imaginary part of permittivity of the 3 mm thick PANI/TiO_2_ and PANI/MoS_2_/TiO_2_ composites.

Nevertheless, at 30 wt % MoS_2_ (PTM3–3), the SE_T_ reached 47.21 dB, indicating a plateau in shielding effectiveness. The excessive MoS_2_ content likely caused the agglomeration and disrupted material uniformity, diminishing overall dielectric efficiency. This observed saturation in SE_T_ aligns with Debye theory, which proposes that beyond a critical concentration of conductive filler, further shielding improvements become negligible due to nonuniform particle distribution.

The power coefficient values for the PTM2–3 sample, calculated using eqs 2 and 6, are shown in Figure 10, revealing a transmission close to zero, a reflection coefficient of approximately 0.7, and an effective absorption coefficient near 0.3, indicating that it absorbs 30% of the incoming electromagnetic energy. With a reflection coefficient of 0.7, the sample demonstrates a strong capacity to reflect 70% of the incident energy. Consequently, the *A*_eff_ of the PMT2–3 sample approaches 1, demonstrating its effectiveness in absorbing and reflecting the majority of incoming electromagnetic energy. The combination of high reflection and absorption, coupled with minimal transmission, substantially enhances the material’s EMI shielding effectiveness, making it highly suitable for applications requiring electromagnetic wave attenuation.

**Figure 10:**
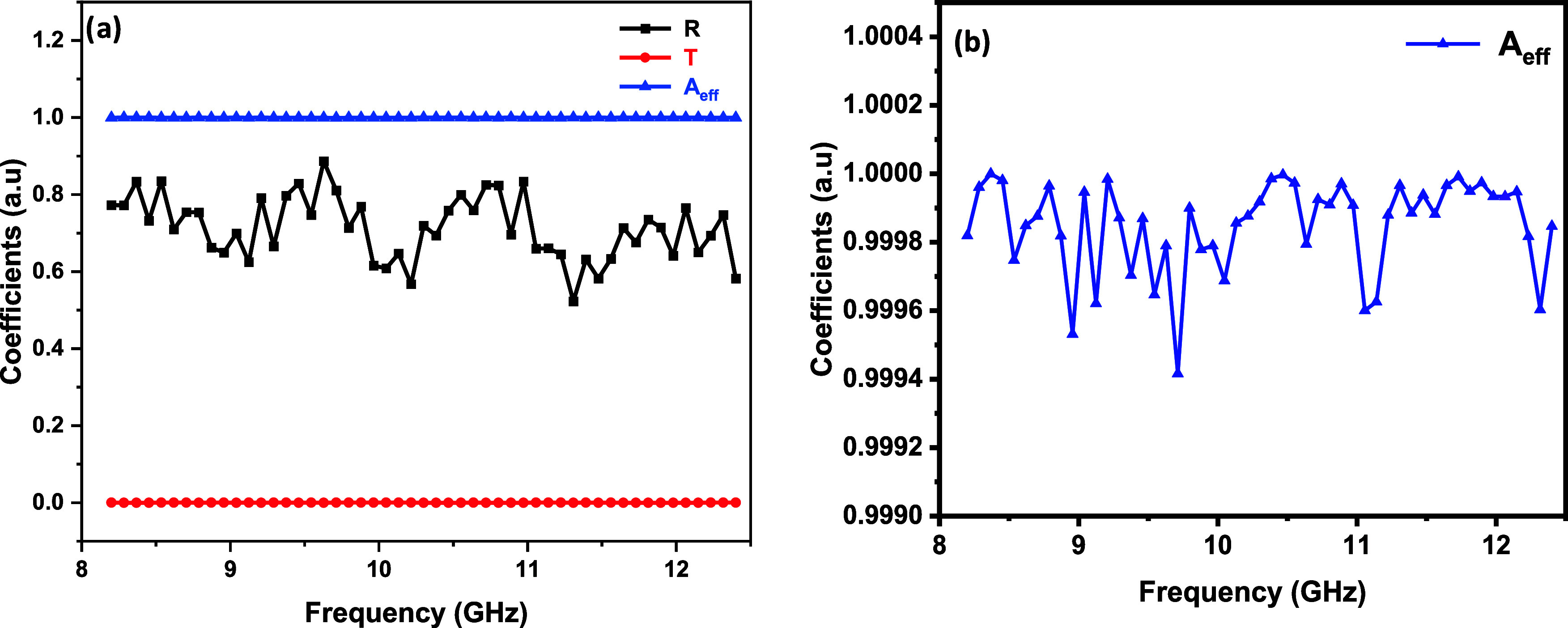
(a) Reflection, transmission, and effective absorption coefficients of the PTM2–3 sample, (b) Close view of *A*_eff_ data to highlight variations along the *x*-axis.

To assess the performance of the PANI/TiO_2_/MoS_2_ composite further, Table 1 presents a comparison of its SE with other recently reported EMI shielding materials that utilize MXenes and graphene. The findings reveal that our ternary composite offers competitive shielding capabilities, especially given its moderate thickness and the lack of costly or environmentally harmful fillers. The combination of synergistic dielectric and conductive losses allows this composite to achieve a high EMI SE, making it ideal for practical applications requiring lightweight shielding.

**Table 1:** Comparison of EMI Shielding Effectiveness of Various PANI-Based Composites

composite materials	frequency range (GHz)	EMI SE (dB)	refs
PANI/MWCNT/MoS_2_	8.2–12.4	48.98	^22^
MWCNT/Graphene/Polyaniline	8.2–12.4	78.00	^40^
Ti_3_C_2_T_*x*_/c-PANI	8.2–12.4	36.00	^41^
PANI/NFO@RGO	8.2–12.4	31.15	^42^
MXene/PANI/aramid	8.2–12.4	31.87	^43^
Fe_3_O_4_/rGO/PANI/Ni–P	8.2–12.4	81.00	^44^
TiO_2_/MXene	8.2–12.4	73.98	^45^
PANI/TiO_2_/MoS_2_	8.2–12.4	50.86	this work

Incorporating MoS_2_ into PANI/TiO_2_ composites significantly enhances their SE_T_ by improving dielectric polarization and energy dissipation. The observed SE_T_ value trends can be explained using Debye theory, which highlights the significance of both real and imaginary permittivity in determining the dielectric responses of materials. The composite achieves an optimal balance of dielectric properties at approximately 20 wt % MoS_2_ concentration, leading to superior EMI shielding. This enhancement is attributed to increased polarization and energy dissipation, as predicted by Debye theory, with real permittivity increasing in proportion to MoS_2_ concentration and imaginary permittivity showing a significant rise at higher frequencies, indicating improved dielectric losses and shielding effectiveness.

However, the benefits of MoS_2_ begin to diminish beyond a concentration of 30 wt % due to particle agglomeration, which negatively impacts the composite’s homogeneity and performance. This results in a plateau of SE_T_ values, as demonstrated in the 3 mm thick samples, where further increases in MoS_2_ content produce diminishing returns. The improved dielectric behavior observed up to 20 wt % MoS_2_ supports the concept of enhanced polarization and dissipation, but once MoS_2_ reaches its critical concentration, excessive loading hinders the composite’s ability to further improve its dielectric properties. This phenomenon, explained by Debye theory, emphasizes the importance of a well-balanced MoS_2_ concentration to maximize both the shielding performance and dielectric behavior of the composite.

The total SE_T_ of the PANI/TiO_2_/MoS_2_ composites improved consistently across all MoS_2_ concentrations as the thickness increased. This improvement is due to greater attenuation of electromagnetic waves through both absorption and multiple reflections within the thicker material layers. Samples with increased thickness provide a longer path for wave dissipation, enhancing the interaction between incident electromagnetic waves and the composite’s dielectric medium. At higher MoS_2_ concentrations, where the agglomeration reduces the dielectric efficiency, wider thickness compensated for these limitations by enhancing wave attenuation. Consequently, optimizing both MoS_2_ concentration and composite thickness is crucial for achieving superior EMI shielding performance.

## Conclusions

We have successfully synthesized and characterized PANI/TiO_2_/MoS_2_ composites for EMI shielding applications, utilizing in situ polymerization of PANI along with TiO_2_ and MoS_2_. The composites were synthesized for various concentrations of MoS_2_ and systematically evaluated for their shielding performance over the X-band frequency range (8.2–12.4 GHz) using a waveguide system and VNA. Our findings revealed that MoS_2_ concentration significantly affects the SE of these composites. Optimal performance was achieved with 20 wt % MoS_2_ concentration and a thickness >1.5 mm, resulting in SE_T_ values up to 50.86 dB. This enhanced SE is due to the combined effects of MoS_2_, PANI, and TiO_2_, where MoS_2_ incorporation boosts both dielectric polarization and energy dissipation, leading to improved shielding performance. Interestingly, increasing MoS_2_ concentration beyond this optimal point (e.g., to 30 wt %) led to decreased SE. This reduction in SE can be explained by particle agglomeration occurring at higher MoS_2_ concentrations, which disrupts conductive pathways and reduces composite uniformity. Consequently, the material’s overall conductivity decreases, impeding electron transport crucial for effective EMI shielding. These observations align with Debye theory, which emphasizes the crucial balance between conductivity and dielectric loss in determining the EMI shielding capabilities of composite materials. Based on these results, we suggest that PANI/TiO_2_/MoS_2_ composites are promising candidates for EMI shielding applications across various sectors, including automotive, aerospace, defense, and electronics industries. These materials show particular potential for flexible and thin-film applications. The optimized composition and thickness of the composite material indicate its suitability for high-performance EMI shielding in both portable devices and industrial equipment.

## References

[1] Shahzad F., Alhabeb M., Hatter C. B., Anasori B., Hong S. M., Koo C. M., Gogotsi Y. (2016). Electromagnetic
interference shielding
with 2D transition metal carbides (MXenes). Science.

[2] Saini, P. Thermoset Nanocomposites; John Wiley & Sons, Ltd, 2013; Chapter 10, pp 211–237.

[3] Shukla V. (2019). Review of
electromagnetic interference shielding materials fabricated by iron
ingredients. Nanoscale Adv..

[4] Wan Y.-J., Zhu P.-L., Yu S.-H., Sun R., Wong C.-P., Liao W.-H. (2017). Graphene paper for exceptional EMI
shielding performance
using large-sized graphene oxide sheets and doping strategy. Carbon.

[5] Gairola S., Verma V., Singh A., Purohit L., Kotnala R. (2010). Modified composition
of barium ferrite to act as a microwave absorber in X-band frequencies. Solid State Commun..

[6] Goel S., Garg A., Baskey H. B., Pandey M. K., Tyagi S. (2021). Studies on
dielectric and magnetic properties of barium hexaferrite and bio-waste
derived activated carbon composites for X-band microwave absorption. J. Alloys Compd..

[7] Moseley, H. Handbook of Laboratory Health and Safety Measures; Pal, S. B. , Ed.; Springer Netherlands: Netherlands, 1990; pp 397–426.

[8] Wang D., Weyen D., Van Tichelen P. (2023). Proposals
for Updated EMC Standards
and Requirements (9–500 kHz) for DC Microgrids and New Compliance
Verification Methods. Electronics.

[9] Kim B.-J., Bae K.-M., Lee Y. S., An K.-H., Park S.-J. (2014). EMI shielding
behaviors of Ni-coated MWCNTs-filled epoxy matrix nanocomposites. Surf. Coat. Technol..

[10] Saini P., Choudhary V., Vijayan N., Kotnala R. K. (2012). Improved Electromagnetic
Interference Shielding Response of Poly­(aniline)-Coated Fabrics Containing
Dielectric and Magnetic Nanoparticles. J. Phys.
Chem. C.

[11] Zhang Y., Qiu M., Yu Y., Wen B., Cheng L. (2017). A Novel Polyaniline-Coated
Bagasse Fiber Composite with Core-Shell Heterostructure Provides Effective
Electromagnetic Shielding Performance. ACS Appl.
Mater. Interfaces.

[12] Feng A., Jia Z., Zhao Y., Lv H. (2018). Development of Fe/Fe3O4@C composite
with excellent electromagnetic absorption performance. J. Alloys Compd..

[13] Esawi A. M., Farag M. M. (2007). Carbon nanotube reinforced composites:
potential and
current challenges. Mater. Design.

[14] Kumar D. A., Murugesan M. (2024). Design and
analysis of the polypyrrole (PPy) composites
for electromagnetic compatibility. J. Polym.
Res..

[15] Rosarian
Joy S J., Rajan Babu D. (2025). Study of microwave absorption properties
of strontium hexaferrite (SrFe12O19) and impure activated carbon composite
in X-band range (8.2 - 12.4 GHz). Results Phys..

[16] Cho H.-S., Kim S.-S. (1999). M-hexaferrites with
planar magnetic anisotropy and
their application to high-frequency microwave absorbers. IEEE Trans. Magn..

[17] Haijun Z., Zhichao L., Chengliang M., Xi Y., Liangying Z., Mingzhong W. (2002). Complex permittivity, permeability,
and microwave absorption
of Zn-and Ti-substituted barium ferrite by citrate sol-gel process. Mater. Sci. Eng.: B.

[18] Shin J., Oh J. (1993). The microwave absorbing phenomena
of ferrite microwave absorbers. IEEE Trans.
Magn..

[19] Singh P., Babbar V., Razdan A., Srivastava S., Goel T. (2000). Microwave absorption studies of Ca-NiTi
hexaferrite composites in
X-band. Mater. Sci. Eng.: B.

[20] Olmedo, L. ; Hourquebie, P. ; Jousse, F. Handbook of Organic Conductive Molecules and Polymers John Wiley and Sons Ltd: New York, 1997.

[21] Aka C., Akgöl O., Karaaslan M., Akyol M. (2023). Broadband electromagnetic
wave absorbing via PANI coated Fe3O4 decorated MoS2 hybrid nanocomposite. J. Alloys Compd..

[22] Dhanasekaran A., Dhanaraj K., Venugopal V. (2024). Polyaniline/MoS2/MWCNT
Ternary Nanocomposite
for Effective Electromagnetic Interference Shielding. ACS Appl. Nano Mater..

[23] Rao C. N. R., Gopalakrishnan K., Maitra U. (2015). Comparative study of
potential applications of graphene, MoS2, and other two-dimensional
materials in energy devices, sensors, and related areas. ACS Appl. Mater. Interfaces.

[24] Phang S. W., Tadokoro M., Watanabe J., Kuramoto N. (2008). Microwave absorption
behaviors of polyaniline nanocomposites containing TiO2 nanoparticles. Curr. Appl. Phys..

[25] Ma J., Ren H., Liu Z., Zhou J., Wang Y., Hu B., Liu Y., Kong L. B., Zhang T. (2020). Embedded MoS2-PANI
nanocomposites
with advanced microwave absorption performance. Compos. Sci. Technol..

[26] Zhang W. L., Jiang D., Wang X., Hao B. N., Liu Y. D., Liu J. (2017). Growth of Polyaniline
Nanoneedles on MoS2 Nanosheets, Tunable Electroresponse,
and Electromagnetic Wave Attenuation Analysis. J. Phys. Chem. C.

[27] Tang Q., Chen H., Shen J. (2023). Flower-like MoS_2_/cotton
fiber-derived TiO_2_ composites with strong electromagnetic
wave absorption performance. J. Mater. Sci..

[28] Xu X., Wang Y., Yue Y., Wang C., Xu Z., Liu D. (2022). In-situ growth of TiO2
nanoparticles on crumpled Ti3C2Tx with negative
permittivity for electromagnetic interference shielding. Ceram. Int..

[29] Ibrahim K. A. (2017). Synthesis
and characterization of polyaniline and poly­(aniline-co-o-nitroaniline)
using vibrational spectroscopy. Arabian J. Chem..

[30] Wang N., Li J., Lv W., Feng J., Yan W. (2015). Synthesis of polyaniline/TiO_2_ composite with excellent adsorption performance on acid red
G. RSC Adv..

[31] Huyen D. N., Tung N. T., Thien N. D., Thanh L. H. (2011). Effect of TiO2 on
the Gas Sensing Features of TiO2/PANi Nanocomposites. Sensors.

[32] El-Deen S. S., Hashem A. M., Ghany A. E. A., Indris S., Ehrenberg H., Mauger A., Julien C. M. (2018). Anatase TiO_2_ nanoparticles
for lithium-ion batteries. Ionics.

[33] El-Desoky M. M., Morad I., Wasfy M. H., Mansour A. F. (2020). Synthesis, structural
and electrical properties of PVA/TiO_2_ nanocomposite films
with different TiO_2_ phases prepared by sol-gel technique. J. Mater. Sci.: Mater. Electronics.

[34] Raghu M. S., Kumar K. Y., Rao S., Aravinda T., Prasanna B. P., Prashanth M. K. (2018). Fabrication
of polyaniline-few-layer MoS_2_ nanocomposite for high energy
density supercapacitors. Polym. Bull..

[35] Ghaleghafi E., Rahmani M. B. (2022). Exploring different
routes for the synthesis of 2D
MoS2/1D PANI nanocomposites and investigating their electrical properties. Phys. E.

[36] Zhao B., Yan Z., Liu L., Zhang Y., Guan L., Guo X., Li R., Che R., Zhang R. (2024). A Liquid Metal Assisted Competitive
Galvanic Reaction Strategy Toward Indium/Oxide Core Shell Nanoparticles
with Enhanced Microwave Absorption. Adv. Funct.
Mater..

[37] Che R., Peng L.-M., Duan X., Chen Q., Liang X. (2004). Microwave
Absorption Enhancement and Complex Permittivity and Permeability of
Fe Encapsulated within Carbon Nanotubes. Adv.
Mater..

[38] Omana L., Chandran A., John R. E., Wilson R., George K. C., Unnikrishnan N. V., Varghese S. S., George G., Simon S. M., Paul I. (2022). Recent Advances in Polymer Nanocomposites for Electromagnetic Interference
Shielding: A Review. ACS Omega.

[39] Guo Y., Zhao W., Li D., Liu J., Qian J., Pang L., Zhou T., Liu W., Liu Z., Huang H., Zhai J., Zhou D. (2025). Ultra-High Capacitive
Energy Storage Density at 150 °C Achieved in Polyetherimide Composite
Films by Filler and Structure Design. Adv. Mater..

[40] Xie Z., Chen H., Xie M., Zhang D., Zhao H., Chen W. (2024). Electrical percolation networks of MWCNT/Graphene/Polyaniline nanocomposites
with enhanced electromagnetic interference shielding efficiency. Appl. Surf. Sci..

[41] Zhang Y., Wang L., Zhang J., Song P., Xiao Z., Liang C., Qiu H., Kong J., Gu J. (2019). Fabrication
and investigation on the ultra-thin and flexible Ti3C2Tx/co-doped
polyaniline electromagnetic interference shielding composite films. Compos. Sci. Technol..

[42] Kazmi S. J., Rehman S. U., Nadeem M., Rehman U. U., Hussain S., Manzoor S. (2024). Effect of carbon allotropes
and thickness variation
on the EMI shielding properties of PANI/NFO@CNTs and PANI/NFO@RGO
ternary composite systems. Phys. Chem. Chem.
Phys..

[43] Li X., Liu M., Fang Y., Wu Z., Dong J., Zhao X., Teng C. (2024). Flexible and efficient
MXene/PANI/aramid fabrics with high interface
durability for wearable electromagnetic wave shielding. Composites Commun..

[44] Sedighi A., Naderi M., Brycki B. (2023). Wearable nonwoven fabric
decorated
with Fe3O4/rGO/PANI/Ni-P for efficient electromagnetic interference
shielding. J. Alloys Compd..

[45] Xu X., Wang Y., Yue Y., Wang C., Xu Z., Liu D. (2022). In-situ growth of TiO2
nanoparticles on crumpled Ti3C2Tx with negative
permittivity for electromagnetic interference shielding. Ceram. Int..

